# *TACSTD2* upregulation is an early reaction to lung infection

**DOI:** 10.1038/s41598-022-13637-9

**Published:** 2022-06-10

**Authors:** Sára Lenárt, Peter Lenárt, Lucia Knopfová, Hana Kotasová, Vendula Pelková, Veronika Sedláková, Ondřej Vacek, Jana Pokludová, Vladimír Čan, Jan Šmarda, Karel Souček, Aleš Hampl, Petr Beneš

**Affiliations:** 1 Department of Experimental Biology, Faculty of Science, Masaryk University, Kamenice 5, Brno, 62500 Czech Republic; 2 Faculty of Science, Research Centre for Toxic Compounds in the Environment, Masaryk University, Brno, Czech Republic; 3 Institute of Cell Biology, University of Bern, Bern, Switzerland; 4 International Clinical Research Center, St. Anne’s University Hospital, Brno, Czech Republic; 5 Department of Histology and Embryology, Faculty of Medicine, Masaryk University, Brno, Czech Republic; 6 Department of Surgery, University Hospital Brno, Brno, Czech Republic; 7 Department of Cytokinetics, Institute of Biophysics of the Czech Academy of Sciences, Brno, Czech Republic

**Keywords:** Cell biology, Developmental biology, Molecular biology, Molecular medicine

## Abstract

*TACSTD2* encodes a transmembrane glycoprotein Trop2 commonly overexpressed in carcinomas. While the Trop2 protein was discovered already in 1981 and first antibody–drug conjugate targeting Trop2 were recently approved for cancer therapy, the physiological role of Trop2 is still not fully understood. In this article, we show that *TACSTD2*/Trop2 expression is evolutionarily conserved in lungs of various vertebrates. By analysis of publicly available transcriptomic data we demonstrate that *TACSTD2* level consistently increases in lungs infected with miscellaneous, but mainly viral pathogens. Single cell and subpopulation based transcriptomic data revealed that the major source of *TACSTD2* transcript are lung epithelial cells and their progenitors and that *TACSTD2* is induced directly in lung epithelial cells following infection. Increase in *TACSTD2* expression may represent a mechanism to maintain/restore epithelial barrier function and contribute to regeneration process in infected/damaged lungs.

## Introduction

Trophoblast cell surface antigen 2 (Trop2) is a transmembrane glycoprotein with yet unresolved physiological function, that is overexpressed in most carcinomas where it has been associated with cancer cell plasticity, tumor growth, metastasis and prognosis^1,2^. It is encoded by the intronless *TACSTD2* (tumor-associated calcium signal transducer 2) gene belonging to *TACSTD* gene family^3^. Genes of the *TACSTD* gene family are highly conserved across species; for instance, mouse Trop2 is 79.2% identical and 87.4% similar to human Trop2^4,5^. Trop2 was originally found on the surface of trophoblast cells^6^ and has been subsequently identified on healthy epithelial cells of various other organs^7,8^. Trop2 is also expressed during normal embryonal and fetal development in lungs^9,10^, intestines^11^, stomach^12^, urinary bladder^13^, kidneys^14^, and cerebellum^15^, however, its function in healthy adult tissues remains unknown.

In humans, congenital mutations of *TACSTD2* cause a gelatinous drop-like corneal disease (GDLD), a rare autosomal recessive disease characterized by the development of bilateral corneal amyloidosis and eventually blindness^16^. Loss of the Trop2 function leads to impaired subcellular localizations of tight junction-related proteins and loss of barrier function of corneal epithelial cells resulting in passage of lactoferrin to subepithelial region where it forms amyloid deposits^17^. Trop2 is also considered to be a stem/progenitor cell marker^11,13,18–21^ and several studies indicate that it might be associated with tissue remodeling and regeneration processes^12,22,23^. Surprisingly, the *Tacstd2* null mice are fully viable, fertile, and without overt developmental abnormalities^24^.

Lungs are vital organs inherently vulnerable to infection and injury due to constant exposure to pathogens, chemicals, and other air pollutants. The proper functions of epithelial barrier, immune system, and regenerative capacity of the lungs are thus crucial for restoring homeostasis following pathogen exposure or acute injury^25^. The importance of lung homeostasis maintenance is further highlighted by the fact that even before the rise of SARS-CoV-2 pandemic, respiratory diseases belonged to leading causes of death worldwide^26^. In this study, we use available expression datasets to test the hypothesis that the upregulation of *TACSTD2* in the lungs is a physiological reaction to infection or injury, which both trigger an acute immune response^27–29^.

## Results

### *TACSTD2*/Trop2 expression in lungs

To test the hypothesis that upregulation of *TACSTD2*/Trop2 is a physiological reaction to lung tissue damage by infection or injury, we first verified that *TACSTD2* is expressed in healthy lungs indeed. Analysis of available datasets shows overwhelming evidence that the *TACTSD2* gene is expressed in lungs of all studied species (Table 1). This suggests that *TACSTD2* has an evolutionarily conserved role in the lung function. A more detailed table is available in Supplementary File 1.

**Table 1:** List of studied organisms with *TACSTD2* expression in lungs.

Organism	Number of subjects
Human (*Homo sapiens*)	601/601*
Mouse (*Mus musculus*)	27/27*
Cattle (*Bos taurus*)	4/4
Chicken (*Gallus gallus*)	3/3
Sheep (*Ovis aries*)	4/4
Olive baboon (*Papio anubis*)	1/1
Rat (*Rattus norvegicus*)	35/35
Pig (*Sus scrofa*)	2/4
Chimpanzee (*Pan troglodytes*)	1/1
Macaque (*Macaca mulata*)	1/1
Rabbit (*Oryctolagus cuniculus*)	1/1
Opossum (*Monodelphis domestica*)	1/1

The number of subjects reflects the sum of biological replicates from transcriptomic datasets.

*Trop2 expression was also found in human lungs in two out of three proteomics datasets. The level of mouse Trop2 was below the cutoff in the one available proteomic dataset (for detailed informations about all transcriptomic and proteomic datasets see Supplementary File 1).

**Figure 1:**
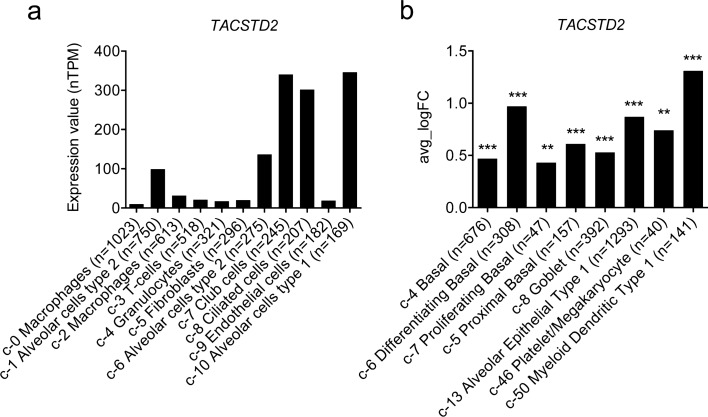
*TACSTD2* expression in cell clusters of human lungs identified by single cell RNA sequencing. (**a**) RNA expression (nTPM) in the cell type clusters identified in lungs visualized by a bar chart, retrieved from Human Protein Atlas. Single cell transcriptomic dataset of Vieira Braga *et al*. (GSE130148)^30^ was used. (**b**) Cell clusters with significantly (p adj < 0.05) enriched *TACSTD2* expression as identified in human lungs by Travaglini *et al*.^31^. Chart shows the natural log of the average fold change between the indicated cell type and other cell types in lungs. Differentially expressed genes were identified using the ‘MAST’ statistical framework implemented in Seurat’s ‘FindMarkers’ function by Travaglini *et al*.^31^. *c* cluster number, with main cell type annotated, *n* number of included cell, **p adj < 0.01, ***p adj < 0.001, *p adj* p-value with Bonferroni correction applied.

To find which cell types produce *TACSTD2* in the lungs, we searched the Human Protein Atlas. The highest expression has been detected in alveolar cells type I and II, club cells and ciliated cells but smaller amounts of *TACSTD2* were also expressed in lung’s immune cells, such as macrophages, T-cells, and granulocytes (Fig. 1a)^30^. Recently, single cell transcriptomic analysis revealed that out of 58 molecular cell types identified in human lungs, *TACSTD2* is enriched in basal, differentiating basal, proliferating basal, proximal basal, goblet, alveolar epithelial type 1, platelets, and myeloid dendritic cells (Fig. 1b)^31^.

**Figure 2:**
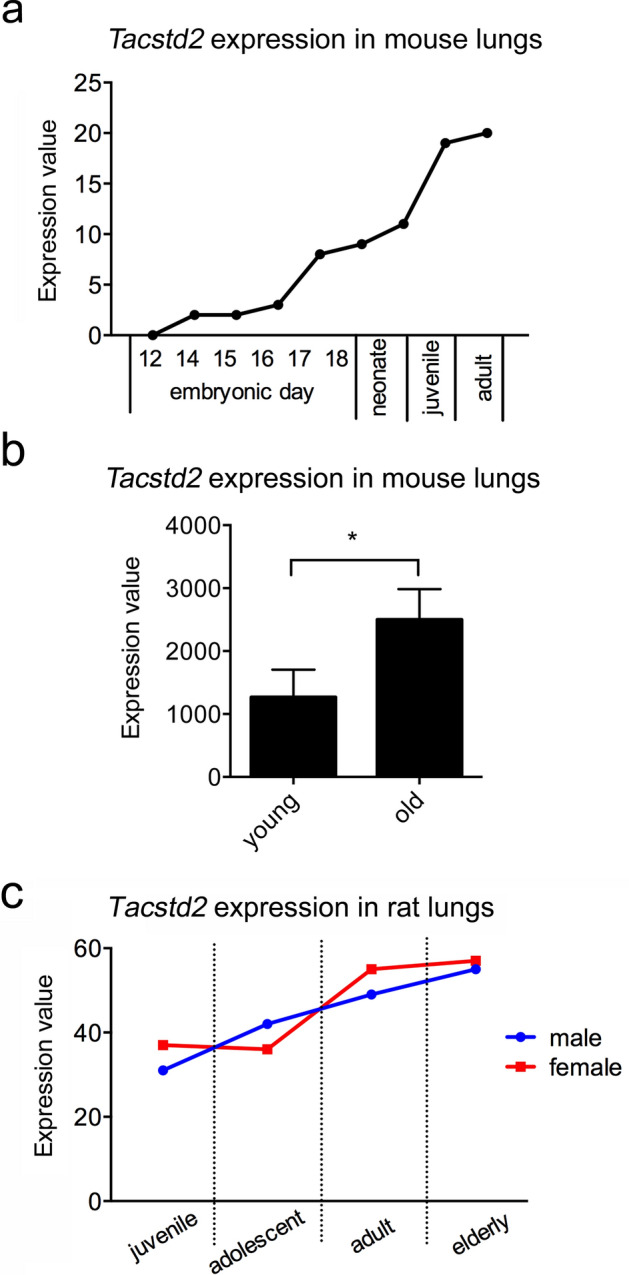
*Tacstd2* expression increases in lungs during embryonic development and ageing. *Tacstd2* gene expression in lungs (bulk data) of (**a**) six mice during embryonal development, five neonate, two juvenile mice and one adult mouse (E-MTAB-3579), expression value represents median TPM. (**b**) Three replicates of young (3 months) and old mice (24 months), expression value represents count per million normalized by voom function of the limma R package^33^. Significant difference (*p < 0.05) is indicated. Data are presented as mean ± SD and were analyzed with unpaired t-test using GraphPad Prism v6.07. (**c**) Juvenile (2 weeks), adolescent (6 weeks), adult (21 weeks) and elderly (104 weeks) female and male rats (E-GEOD-53960), expression value represents median TPM^32^. Four biological replicates were used for each sex and developmental stage.

Interestingly, one mouse (E-MTAB-3579) and one rat (E-GEOD-53960) dataset evaluated transcripts at different stages of embryonal development and at different stages of postnatal life^32^. In these datasets, *Tacstd2* expression increased with age (Fig. 2a,c). This has been recently confirmed by Angelidis *et al*., who detected significantly higher *Tacstd2* mRNA in the bulk lung RNA of 24-months-old mice than in the lungs of 3-months-old mice (Fig. 2b). Single cell transcriptomic approach, however, did not reveal the source of this increase^33^.

**Figure 3:**
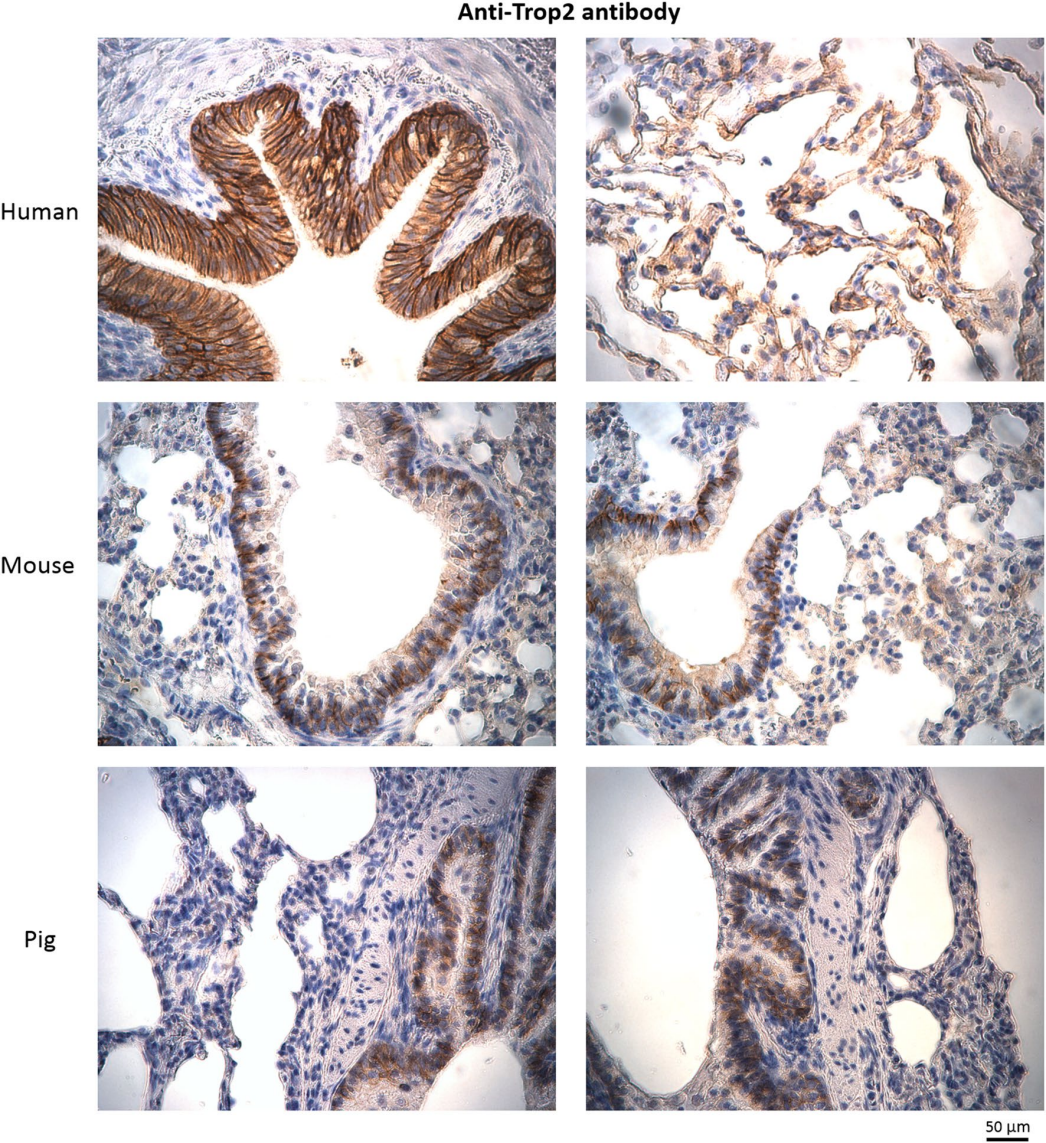
Immunohistochemical detection of Trop2 in paraffin sections of human, mouse and pig lung tissue. Human lungs—positive staining in epithelium of airway and alveoli. Mouse/Pig lungs—positive staining only in basolateral parts of airway epithelium.

To confirm that Trop2 protein is expressed in lungs of various organisms we performed immunohistochemical analysis in paraffin sections of human, mouse and pig lung tissues. In human lungs, Trop2 staining was observed in membranes of airway and alveolar epithelial cells while only basolateral parts of airway epithelium was positive for Trop2 in mouse and pig lungs (Fig. 3, Supplementary File 2, 3). These data confirm that Trop2 is produced by lung epithelial cells but also point to differences in its expression pattern in lungs of selected vertebrates.

### Upregulation of *TACSTD2* in infected lungs

**Figure 4:**
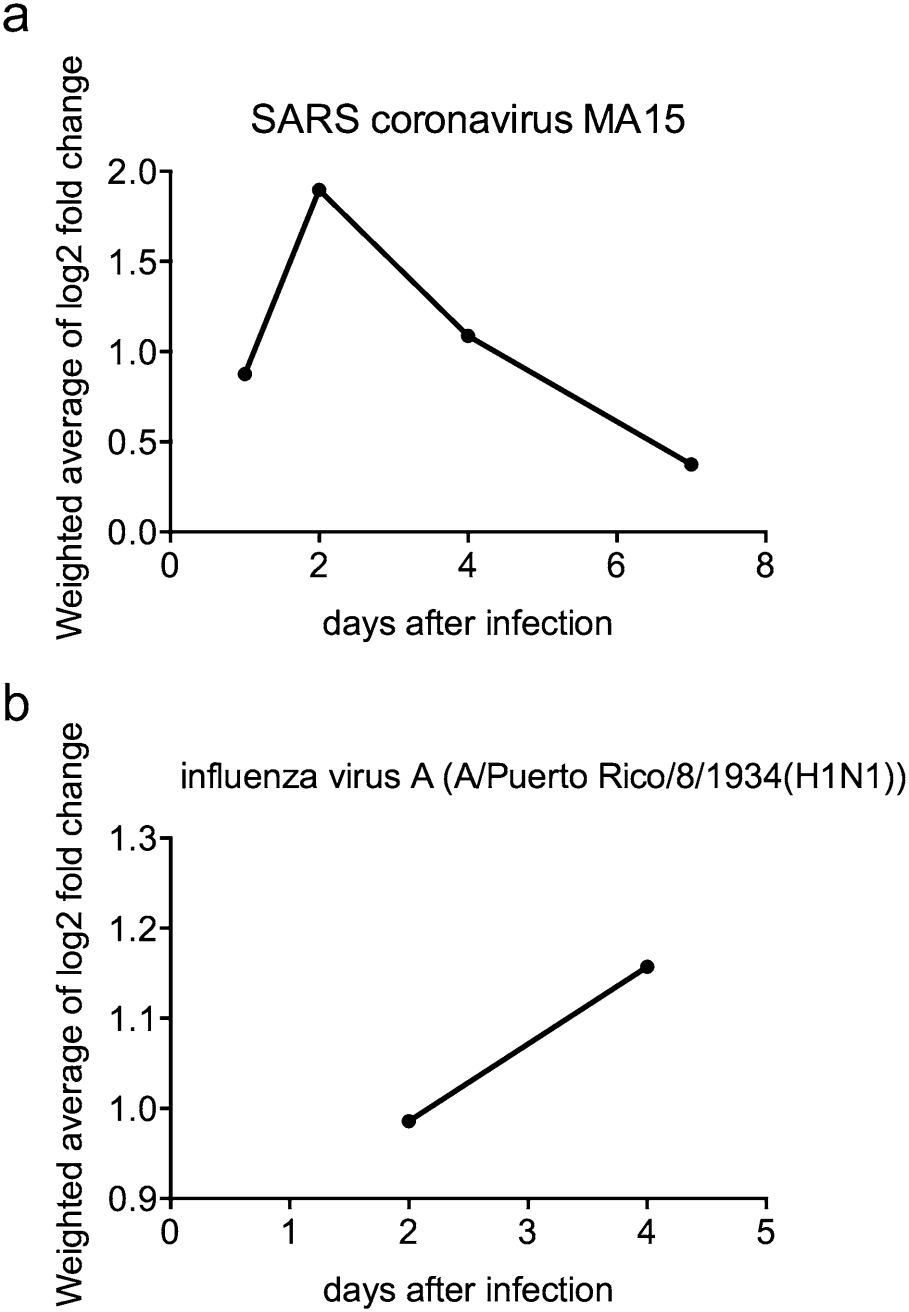
Increase in *Tacstd2* expression is an early reaction to infection with SARS coronavirus MA15 and influenza A virus. Weighted average of *Tacstd2* log_2_-fold change after infection of mice with (**a**) SARS coronavirus MA15 (n = 155) and (**b**) influenza A virus (n = 42) at different time points. Only data from mice infected with 10^5^ PFU in case of SARS coronavirus and 10^2^ PFU in case of influenza A virus are shown. Data from infection with mutant viruses were excluded.

Next, to analyze *TACSTD2* expression in response to lung damage, we searched the Expression Atlas database for differential expression in lungs after infection or injury. Eleven differential expression datasets analyzed the levels of *Tacstd2* early after infection (1–7 days), and all of them showed a significant upregulation of *Tacstd2* in infected mouse lungs (Table 2). This increase was observed in both males and females, 9 different mouse strains, at various ages (8–20 weeks), and with various infectious agents including SARS coronavirus, influenza A virus, Sendai virus and *Mycobacterium tuberculosis*. Datasets containing information about the dynamics of this process showed that in the case of SARS coronavirus MA15, the levels of *Tacstd2* peaked two days post-infection and then started to decrease, indicating that the upregulation of *Tacstd2* is an early reaction to infection (Table 2, Fig. 4a). After infection with the influenza A virus, *Tacstd2* levels were elevated on the fourth day by approximately 20% (Table 2, Fig. 4b). Particularly intriguing is the E-GEOD-33266 dataset, where various doses of SARS coronavirus were used for infecting mice. Analysis of this dataset not only confirmed that *Tacstd2* levels were highest two days after SARS coronavirus infection regardless of the infection dose but also showed that a higher dose of the virus induced higher upregulation of *Tacstd2* (Table 2).

**Table 2:** Differential *Tacstd2* expression in mice after infection with various pathogens.

ArrayExpress accession number	Infect	Time (days)	Log_2_-fold change	Adjusted p-value	Number of subjects	Strain	Age (weeks)	Sex
E-GEOD-49262	SARS coronavirus MA15 dORF6 vs mock	1	0.1	0.423	3 vs 3	C57BL/6J	20	Mixed
		2	2.4	** < 0.001**	3 vs 3			
		4	1.2	**0.001**	3 vs 3			
		7*	1.2	N/A	3 vs 2			
	SARS coronavirus MA15 vs mock	1	1.3	**0.001**	3 vs 3	C57BL/6J	20	Mixed
		2	2.3	** < 0.001**	3 vs 3			
		4	1	**0.002**	3 vs 3			
		7*	1.4	N/A	3 vs 2			
E-GEOD-49263	SARS coronavirus MA15 nsp16−/− vs mock	1*	1.3	N/A	3 vs 2	C57BL/6J	10	Mixed
		2	2.3	** < 0.001**	4 vs 3			
		4	0.9	**0.006**	3 vs 3			
		7	0.1	0.747	4 vs 3			
	SARS coronavirus MA15 vs mock	1*	1.4	N/A	4 vs 2	C57BL/6J	10	Mixed
		2	2.4	** < 0.001**	4 vs 3			
		4	1.2	** < 0.001**	4 vs 3			
		7	0.5	0.055	3 vs 3			
E-GEOD-50878	SARS coronavirus MA15 vs mock	2	2	** < 0.001**	3 vs 9	C57BL/6J	10	Not available
		4*	1.1	N/A	2 vs 9			
		7	0.3	**0.040**	3 vs 9			
	SARS coronavirus MA15 vs mock	2	2.2	** < 0.001**	3 vs 7	C57BL/6J CXCR3 knockout	10	Not available
		4*	1.2	N/A	2 vs 7			
		7	0.4	0.146	4 vs 7			
E-GEOD-52405	SARS coronavirus MA15 vs mock	2	1.5	** < 0.001**	3 vs 4	129S1/SvImJ	8 to 16	Female
		4	0.7	** < 0.001**	3 vs 4			
		2	2	** < 0.001**	3 vs 4	C57BL/6J	8 to 16	Female
		4	1	** < 0.001**	3 vs 4			
		2	0.9	** < 0.001**	3 vs 4	CAST/EiJ	8 to 16	Female
		4	0.6	0.134	3 vs 4			
		2	1.4	** < 0.001**	3 vs 4	NOD/ShiLtJ	8 to 16	Female
		4	0.8	** < 0.001**	3 vs 4			
		2	1.5	** < 0.001**	3 vs 4	PWK/PhJ	8 to 16	Female
		4	1.8	** < 0.001**	3 vs 4			
		2	1.6	** < 0.001**	3 vs 4	WSB/EiJ	8 to 16	Female
		4	1	** < 0.001**	3 vs 4			
		4	1.7	** < 0.001**	3 vs 4	A/J	8 to 16	Female
	Influenza A virus (A/Puerto Rico/8/1934(H1N1)) (10^2^ PFU) vs mock	2	1.2	** < 0.001**	3 vs 4	129S1/SvImJ	8 to 16	Female
		4	0.9	** < 0.001**	3 vs 4			
		2	1.4	** < 0.001**	3 vs 4	A/J	8 to 16	Female
		4	0.9	**0.002**	3 vs 4			
		2	1.8	** < 0.001**	3 vs 4	NOD/ShiLtJ	8 to 16	Female
		4	1.5	** < 0.001**	3 vs 4			
		2	0.7	0.172	3 vs 4	C57BL/6J	8 to 16	Female
		4	1.1	** < 0.001**	3 vs 4			
		2	0.6	0.141	3 vs 4	NZO/HILtJ	8 to 16	Female
		4	1.3	**0.008**	3 vs 4			
		4	1.1	** < 0.001**	3 vs 4	PWK/PhJ	8 to 16	Female
		2	0.8	** < 0.001**	3 vs 4	CAST/EiJ	8 to 16	Female
		2	0.4	0.406	3 vs 4	WSB/EiJ	8 to 16	Female
		4	1.3	** < 0.001**	3 vs 4			
E-GEOD-68820	SARS coronavirus MA15 vs mock	2	2	** < 0.001**	5 vs 4	C57BL/6NJ TLR3 knockout	10	Female
		4	1.2	** < 0.001**	5 vs 4			
		7*	-0.02	N/A	5 vs 2			
		2	2	** < 0.001**	5 vs 5	C57BL/6NJ	10	Female
		4	0.6	** < 0.001**	4 vs 5			
		7	-0.2	0.230	4 vs 4			
E-GEOD-59185	SARS coronavirus MA15 vs mock	2	1.9	** < 0.001**	3 vs 3	BALB/c	16	Female
	SARS coronavirus MA15 E protein mutant Δ3 vs mock	2	1.5	**0.002**	3 vs 3	BALB/c	16	Female
	SARS coronavirus MA15 E protein mutant Δ5 vs mock	2	–	–	3 vs 3	BALB/c	16	Female
	SARS coronavirus MA15 lacking full-length E protein vs mock	2	0.5	0.179	3 vs 3	BALB/c	16	Female
E-MTAB-5218	Mycobacterium tuberculosis H37Rv (1000 ± 300 CFU) vs mock	28	1.6	** < 0.001**	4 vs 3	C57BL/6 TNF-α knockout	8 to 12	Female
		28	–	–	10 vs 9	C57BL/6	8 to 12	Female
E-GEOD-51386	SARS coronavirus MA15 (10^4^ PFU) vs mock	4	1.4	** < 0.001**	4 vs 4	C57BL/6	20	Not available
		7	0.9	** < 0.001**	3 vs 4			
		4	1.1	** < 0.001**	4 vs 4	C57BL/6 PAI1 knockout	20	Not available
		7	0.8	**0.001**	3 vs 4			
		4	1.1	** < 0.001**	4 vs 4	C57BL/6 TIMP1 knockout	20	Not available
		7	0.6	**0.020**	4 vs 4			
E-MTAB-6044	Influenza A virus (500 PFU) vs mock (IgG1 isotype control)	7	1.1	** < 0.001**	4 vs 3	C57BL/6	8 to 10	Male
	Influenza A virus (500 PFU) vs mock (treatment with interleukin-22)	7	1.3	** < 0.001**	4 vs 4	C57BL/6	8 to 10	Male
E-GEOD-51387	SARS coronavirus MA15 vs mock	4	1.2	** < 0.001**	3 vs 4	C57BL/6	20	Not available
		7*	1	N/A	2 vs 4			
		4*	1.5	N/A	2 vs 4	C57BL/6 PLAT knockout	20 weeks	Not available
		7	0.5	** < 0.001**	3 vs 4			
E-GEOD-10964	Active Sendai virus vs UV-inactivated Sendai virus (Affymetrix MOE430A Array)	21	1.2	**0.013**	3 vs 3	C57BL/6J	3 to 5	Male
	Active Sendai virus vs UV-inactivated Sendai virus (Affymetrix Mouse430_2 Array)	49	1	**0.002**	3 vs 3	C57BL/6J	3 to 5	Male
E-GEOD-40824	SARS coronavirus MA15 vs mock	4	1.1	** < 0.001**	3 vs 3	C57BL/6J	10	Female
		7	0.2	0.247	3 vs 3			
		4	1	** < 0.001**	3 vs 3	C57BL/6J Tnfrsf1a/1b knockout	10	Female
		7*	− 0.02	N/A	2 vs 2			
E-GEOD-33266	SARS coronavirus MA15 (10^2^ PFU) vs mock	1	0.3	0.066	5 vs 3	C57BL/6	20	Female
		2	0.2	0.639	5 vs 3			
		4	0.2	0.345	5 vs 3			
		7	− 0.1	0.687	5 vs 3			
	SARS coronavirus MA15 (10^3^ PFU) vs mock	1	0.2	0.208	5 vs 3	C57BL/6	20	Female
		2	1.1	**0.018**	5 vs 3			
		4	0.8	** < 0.001**	5 vs 3			
		7	− 0.1	0.667	5 vs 3			
	SARS coronavirus MA15, (10^4^ PFU) vs mock	1	0.4	**0.017**	5 vs 3	C57BL/6	20	Female
		2	2	** < 0.001**	5 vs 3			
		4	0.8	**0.004**	5 vs 3			
		7	− 0.3	0.366	5 vs 3			
	SARS coronavirus MA15 vs mock	1	0.2	0.126	5 vs 3	C57BL/6	20	Female
		2	2.3	** < 0.001**	5 vs 3			
		4	1.1	** < 0.001**	5 vs 3			
		7	0.5	0.107	5 vs 3			

Where not otherwise specified, viral infection dose was 10^5^ plaque forming units (PFU).

Significant results (adjusted p-value < 0.05) are labeled in bold.

*Means that this entry was completely missing from Expression Atlas and log_2_-fold change was calculated from GEO database data using GEO2R. N/A means that p-value could not be calculated due to small number of subjects.

Interestingly, two datasets tested *Tacstd2* expression after a longer time (Table 2). Lungs of mice infected with *Mycobacterium tuberculosis* H37Rv were analyzed after 28 days. In wild type C57BL/6 mice, there was no difference in *Tacstd2* expression level in comparison to its level prior to infection, which is in agreement with other studies showing that *Tacstd2* is upregulated early after infection. However, when tumor necrosis factor α (TNF-α) knockout (KO) mice were infected, an enhanced bacterial burden, high inflammation, oedema, necrosis and increased *Tacstd2* level were detected in the lungs after 28 days. The second dataset evaluated transcriptome in mice infected with the Sendai virus. Interestingly, in this case *Tacstd2* was also upregulated late post-infection (21 and 49 days). Although, at the first sight, it might seem inconsistent with other studies, mice from this dataset developed chronic airway disease similar to human chronic airway diseases, such as asthma and chronic obstructive pulmonary disease (COPD)^34^. Thus, the long upregulation of *Tacstd2* reflects the known upregulation of *TACSTD2* in human COPD^35^, possibly triggered by chronic lung damage.

Significant upregulation of *TACSTD2* expression was also found in bronchoalveolar lavage cells in patients with transplanted lungs colonized by *Aspergillus fumigatus* (E-MTAB-6040). This dataset did not fulfill our inclusion criteria since it did not provide information about lung transcriptome. However, this result suggests that *TACSTD2* is upregulated after fungal infection as well (Supplementary File 4).

The single differential expression dataset (E-GEOD-19743) containing information about *TACSTD2* levels after an injury did not fulfill inclusion criteria as it studied transcripts in blood and not lungs. However, it is interesting to note that it showed significantly upregulated *TACSTD2* in leukocytes after burn injury in both children (60 subjects) and adults (57 subjects). More details are available in Supplementary File 5. Interestingly, levels of *TACSTD2* were higher in the middle stage (11–49 days) than in early stage (< 11 days) of the healing process after injury.

### *TACSTD2* is upregulated in lung epithelial cells after infection

As mentioned earlier, *TACSTD2* is expressed both in lung epithelial and immune cells. It is not clear if upregulation in infected lungs is caused by increased infiltration of immune cells to the lungs or by a direct upregulation in lung epithelial cells (LECs). In order to clarify this issue, we searched GEO datasets for information about *TACSTD2* expression after infection in specific cell types.

**Figure 5:**
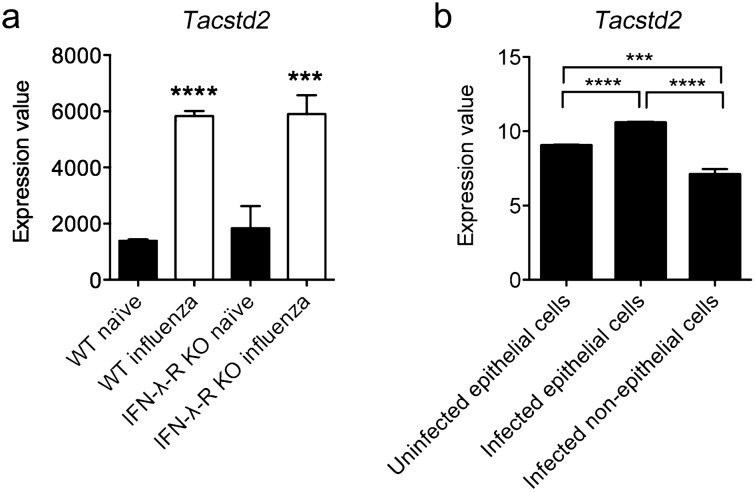
*Tacstd2* expression is increased in lung epithelial cells after infection. *Tacstd2* expression in (**a**) sorted EpCAM^+^CD31^−^CD45^−^ LECs from wt and Ifnlr^−/−^ mice infected or not with influenza virus X31 at 8 days after infection (GSE148709)^36^, shown as normalized counts per gene per sample (generated by DESeq2) and (**b**) sorted EpCAM^+^CD45^−^ LECs from mice infected or not with *Streptococcus pneumoniae* at 15 h after infection (GSE71623), shown as log2-transformed, RMA (Robust Multiarray Average)-normalized gene expression values^37^. Significant differences (***p < 0.001; ****p < 0.0001; unpaired t-test) are indicated.

Datasets examining *Tacstd2* expression in mice infected with influenza virus X31^36^ (GSE148709, Fig. 5a), and *Streptococcus pneumoniae*^37^ (GSE71623, Fig. 5b) showed that *Tacstd2* is significantly upregulated in sorted LECs when compared to uninfected cells. LECs were sorted according to their EpCAM^+^CD31^−^CD45^−^ (GSE148709) or EpCAM^+^CD45^−^ (GSE71623) expression. Upregulation of *Tacstd2* was detected also in LECs of influenza virus X31 infected *Ifnlr1*^*−/−*^ mice (Fig. 5a).

**Figure 6:**
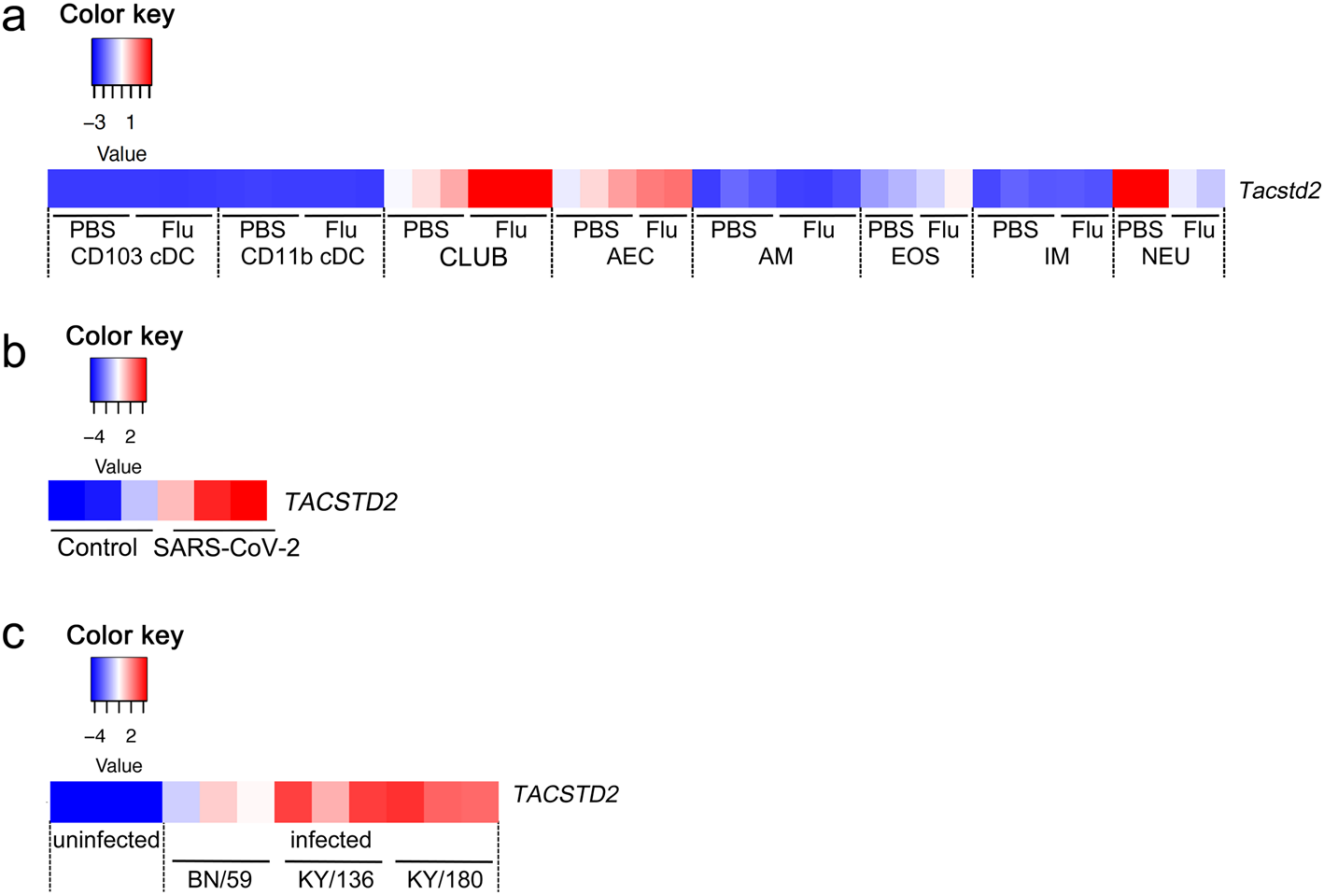
*Tacstd2* expression is increased in lung epithelial cells after infection with influenza virus and SARS-CoV-2. Heat map of *Tacstd2* expression (**a**) in lung resident cells isolated from mouse lungs at 3 days after infection with influenza virus (GSE165299). CD103+ cDC and CD11b+ cDC stands for dendritic cells. CLUB stands for club cells. AEC stands for alveolar epithelial cells. AM stands for alveolar macrophages. IM stands for intersticial macrophages. EOS stands for eosinophils, and NEU stands for neutrophils. Values represent read counts transformed by row scaling (FGCZ heatmap). Heat map of *TACSTD2* expression (**b**) in human alveolar organoids at 48 h after infection with SARS-CoV-2 (GSE152586), values represent FPKM transformed by row scaling (FGCZ heatmap)^38^; (**c**) in human bronchial LECs at 36 h after infection with various influenza A H1N1 isolates (seasonal H1N1 BN/59, pandemic H1N1 KY/136 (non-fatal cases) and pandemic H1N1 KY/180 (fatal cases) (GDS4855), values represent read counts normalized using Gene Console (Affymetrix, version 1.3.1) transformed by row scaling (FGCZ heatmap)^39^.

Recent public dataset GSE165299 contains information about *Tacstd2* expression in mouse lung resident cells. Mice were infected with influenza viruses and 3 days post-infection alveolar epithelial cells (AECs), club cells, dendritic cells (DC), mast cells, macrophages, eosinophils, and neutrophils were isolated and the transcriptome was analyzed. Interestingly, after infection with influenza virus, *Tacstd2* expression was increased in club cells and slightly in AECs and eosinophils, but decreased in neutrophils (Fig. 6a). According to these data neutrophils of uninfected lungs have the highest basal expression of *Tacstd2*.

To further confirm the effect of infection on *TACSTD2* upregulation in lung cells, we searched for datasets examining *TACSTD2* expression in LECs *in vitro*. *TACSTD2* was upregulated in human alveolar type II cell organoids infected with SARS coronavirus 2^38^ (GSE152586, Fig. 6b) as well as in differentiated primary human bronchial LECs infected with various influenza A isolates^39^ (GDS4855, Fig. 6c) when compared to uninfected cells. These data confirm that LECs contribute to the increase of *TACSTD2* levels in lungs following SARS coronavirus and influenza virus infections.

Overall, overexpression of *TACSTD2* is an early event in the lungs challenged with various infection agents. Although increased immune cell infiltration may be partially responsible for this increase, transcriptomic studies in epithelial cells sorted from lungs of infected mice and *in vitro* infected LECs clearly proved direct upregulation of *TACSTD2*.

**Figure 7:**
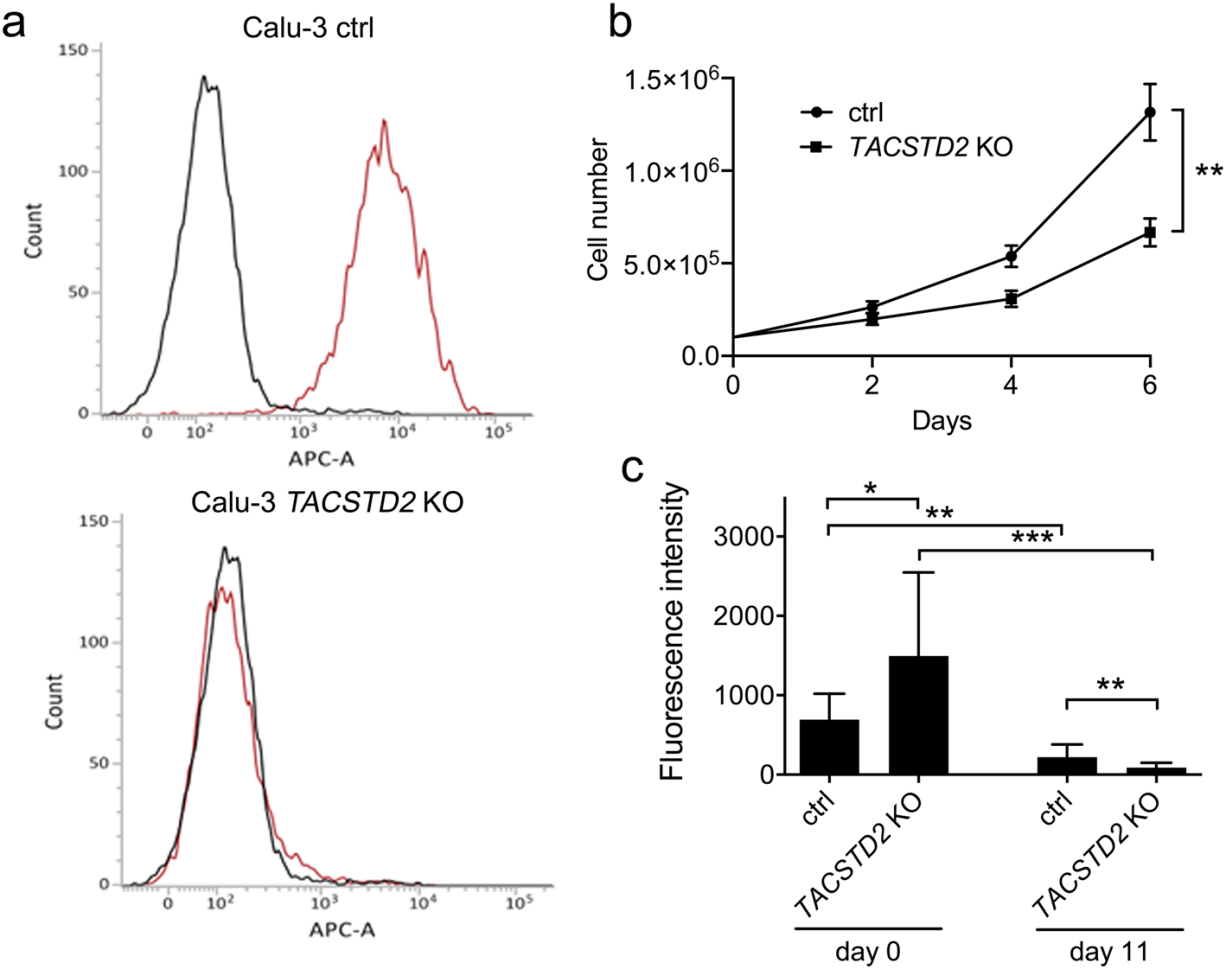
*TACSTD2* KO altered growth and epithelial barrier function in Calu-3 cells. (**a**) Expression of Trop2 in Calu-3 control (ctrl) and *TACSTD2* KO cells determined by flow-cytometry [isotype control IC003A (R&D Systems Inc., Minneapolis, MN) in black, anti-Trop2 antibody FAB650A R&D Systems Inc. in red]. (**b**) Growth curve of control and *TACSTD2* KO cells. (**c**) FITC-dextran flux in control and *TACSTD2* KO cells 3 days after seeding in liquid conditions when cells reached confluence (day 0) and after 11 days of ALI culture (day 11). Significant differences (*p < 0.05, **p < 0.01, ***p < 0.001, Mann–Whitney test) are indicated. Data (**b**,**c**) represents mean ± SD from at least 6 independent experiments.

To analyze the function of Trop2 in LECs, we selected Trop2-expressing Calu-3 lung epithelial cell line as a model^40^. We derived Calu-3 *TACSTD2* KO cells using CRISPR/Cas9 approach (Fig. 7a) and analyzed their growth and epithelial barrier integrity, two processes that may be relevant for LECs response to infection. We observed that *TACSTD2* KO resulted in significant reduction of Calu-3 cell growth (Fig. 7b). To evaluate the epithelial barrier integrity, cells were seeded on Transwell PET membrane inserts and flux of FITC-dextran molecules was evaluated when the cells reached confluence. At this time point, higher flux of FITC-dextran molecules in *TACSTD2* KO cells was observed suggesting worse epithelial barrier function of *TACSTD2* KO cells in comparison with controls (Fig. 7c). When switched to air–liquid interface (ALI) culture, Calu-3 control cells kept growing and overgrew the insert very rapidly while the *TACSTD2* KO cells stayed in monolayer for more than 10 days (Supplementary File 6). Additionally, cultivation in ALI conditions resulted in reduced flux of FITC-dextran molecules in both *TACSTD2* KO and control cells but the decrease was stronger in *TACSTD2* knockouts (Fig. 7c).

## Discussion

In this article, we have analysed available transcriptomic data to decipher the function of *TACSTD2* in lung tissue. Our findings may be summarized as follows. First, we have presented strong evidence that *TACSTD2* is expressed in healthy lungs of various species suggesting its evolutionarily conserved role. Second and most importantly, our analyses have shown that *Tacstd2* is significantly upregulated in mouse lungs following infection with viral respiratory pathogens (SARS coronavirus, influenza virus, Sendai virus), but also non-viral pathogens including *Mycobacterium tuberculosis*, *Streptococcus pneumoniae*, and *Aspergillus fumigatus,* suggesting the involvement of Trop2 in the healing process and/or immune reactions. Additionally, in most datasets, *Tacstd2* overexpression peaks early and decreases over time, suggesting it is an early reaction to infection. In case of chronic inflammation, however, *Tacstd2* remains overexpressed for long time period. The data also show that the level of *Tacstd2* upregulation depend on infection dose. Third, *Tacstd2* upregulation in the lungs after an infection is caused by a direct upregulation in LECs although some contribution of immune cells infiltrating infected lungs cannot be excluded.

Bacterial and viral pathogens are known violators of the airway epithelial barrier’s integrity, which is the first line of defense against infection, decreasing the expression, disrupting or redistributing tight and adherens junctions proteins^41–44^. Such disruption significantly contributes to the pathogenesis of pulmonary infections^43^. Trop2 was previously linked with maintenance of epithelial barrier function in the cornea. *TACSTD2* knockdown in corneal epithelial cells leads to decreased expression and changed subcellular localization of claudin 1, 4, 7, zonula occludens 1, and occludin which results in impaired function of corneal epithelial barrier while transduction of *TACSTD2* restored their expression and epithelial barrier function^45,46^. Decreased expression and altered localization of these proteins were also observed in the corneas of GDLD patients^45^. Moreover, forced expression of *Tacstd2* can at least partially stabilize claudins and restore epithelial barrier in mouse model of congenital tufting enteropathy^47^. To analyze the role of Trop2 in a model of lung epithelial barier, we first screened Trop2 expression in A549, NCI-H23, H1299 (not shown) and Calu-3 lung epithelial cells. From this panel, only Calu-3 cells express Trop2 on cell membrane and were thus used for derivation of *TACSTD2* KO cells. *TACSTD2* KO resulted in higher flux of FITC-dextran in cells cultured on inserts under liquid conditions indicating altered epithielial barrier function in Trop2 KO. The flux was significantly reduced (especially in *TACSTD2* KO cells) when cultured in ALI conditions suggesting that *TACSTD2* KO Calu-3 cells are able to form epithelial barrier in ALI. We are aware of possible limitation of experimental data extrapolation from Calu-3 cells to organ level. It should be noted that Calu-3 cells express high level of EpCAM protein (encoded by *TACSTD1* gene, another *TACSTD* gene family member)^48^. Both proteins share similarities in amino acid sequence, domain structure^49^, processing, cell signaling and protein interaction partners^1,50^. Both proteins interact with tight junction proteins^45,51^ and participate in maintenance of epithelial barrier^46,52,53^. Recent study in *TACSTD1* null mice with forced expression of Trop2 revealed that the function of both proteins is similar but not equivalent^47^. Interestingly, we did not find a similar pattern of deregulation of *TACSTD1* expression in lungs after infection as observed for *TACSTD2* (Supplementary File 7). This finding highlights an important distinction in regulation of expression of both genes and indicates another possible difference in their function. Examination of other models including *TACSTD1* KO and *TACSTD2*/*TACSTD1* double KO cells and thorough inspection of lungs of *Tacstd2* KO mice (and possibly GDLD patients) is thus needed to clarify the exact role of Trop2 in formation of lung epithelial barrier.

It has been shown that Trop2 is expressed in many organs during embryonal development, including lungs^9–15^. It usually marks progenitor cells with high proliferation/self-renewal capacity. *TACSTD2* expression was enhanced during fetal lung expansion and a decreased expression from day 90 of sheep embryonal development to birth was reported corresponding well to decreased rate of cell proliferation (determined by Ki-67 labelling). In rat lungs at E20, about half of Trop2-positive cells were simultaneously positive for Ki-67^9,10^. Based on these data it can be concluded that Trop2 is clearly associated with cell proliferation during lung development. However, although the majority of Ki-67-positive cells were also positive for Trop2, half of the Trop2-positive cells were not positive for Ki-67 suggesting that Trop2 plays other role(s) in lungs. The data on Fig. 2 of our manuscript shows that expression of *Tacstd2* in mouse and rat lungs increases with age suggesting that Trop2 may have other functions in adult lungs that are distinct from its growth-promoting role. Interestingly, recent study identified a significant association between expression of epithelial barrier function genes and age in bronchial brushings of two independent cohorts of healthy individuals suggesting age-related changes in epithelial barrier function. Unfortunately, *TACSTD2* was not in the list of studied genes but *TACSTD1* was the most down-regulated gene in elderly subjects^54^.

The cells exhibiting high Trop2 expression significantly contribute to tissue regeneration in stomach and endometrium^12,22^. In stomach, transcriptome analysis further indicated that Trop2^+^ cells involved in epithelial regeneration overexpress genes that are part of a fetal developmental program^12^. We therefore hypothesize that the upregulation of Trop2 in (sub)population of LECs/progenitor cells may also enhance their proliferative/pro-regenerative capacity and contribute to healing process in infected lungs. It should be noted, however, that the long term Trop2 overexpression associated with inflammation may result in hyperplasia of airway epithelium as observed in lungs of COPD patients^35^. The growth-stimulatory effect of Trop2 was confirmed by us in Calu-3 cells that persisted in ALI conditions as well. This correspond to the results of previous studies in primary lung epithelial cells and lung carcinoma cell lines^35,55^.

Besides the importance of Trop2 for proper localization and function of claudins and occludins in tight junctions and possible pro-regenerative capacity of Trop2-overexpressing cells in airway epithelium, our knowledge about the role of Trop2 in healthy tissues and during infection challenge remains limited. In cancer cells, high-throughput proteomic analysis revealed prosurvival PI3K/Akt as a major cellular signaling pathway stimulated by Trop2^56^. This has been confirmed subsequently in various cancer models^57–60^, and stem cells^21,23^. Activation of the Akt kinase signaling by Trop2 upregulation in response to infection may therefore enhance lung cell survival and decrease tissue damage. It should be noted, however, that controversial role of Akt kinase in modulating infection and inflammation in lungs has been reported^61,62^ vs^63–66^. Interestingly, both Trop2-related functional targets, tight junctions proteins and Akt kinase, were reported to be hijacked by diverse viruses to promote their infection in various tissues. While tight junction proteins may participate in regulation of viral entry, replication, dissemination and progress^67–69^, activation of Akt kinase may represent a strategy of viruses to slow down apoptosis of host cells, thus prolong viral replication and enhance viral transcription^66^. Thorough analysis of *Tacstd2* knockout mice upon infections with various pathogens is therefore needed to help clarify the exact role of the Trop2 protein and its signaling in lung tissue response to infections.

Mechanism of Trop2 increase after infection is currently unknown. However, the early increase of *TACSTD2* expression after infection suggests that innate immunity response may be involved. NF-κB signaling represents a central hub in lung innate immunity response^70^. *TACSTD2* gene was previously identified as NF-κB target in breast cancer^71^ and more recently, NF-κB antagonists inhibited cigarette smoke extract-induced *TACSTD2* expression in airway basal cells^72^. Other transcription factors associated with lung injury/infection and anti-viral response that has been previously identified as *TACSTD2* regulators include CREB and p53^8,71,73–76^. Interestingly, as we observed deregulation of *TACSTD2* expression by SARS coronavirus and influenza A infections in LECs *in vitro*, we suggest that viral transcription machinery and/or cellular anti-viral response (such as p53) may be also involved in *TACSTD2* regulation.

Taken together, using available transcriptomic datasets we demonstrate that *TACSTD2* expression is evolutionarily conserved in the lungs of vertebrates and that the major source of *TACSTD2* transcript are lung epithelial cells and their progenitors. We found that lung levels of *TACSTD2* consistently increase as an early reaction to infection with various (mainly viral) respiratory pathogens. Although this increase may represent a mechanism to maintain/restore epithelial barrier function and to mark pro-regenerative activation of progenitor cells in infected lungs, further studies are needed to clarify the exact role of the Trop2 protein and its signaling during course of lung infections and healing process.

## Methods

### Lung expression data analysis

In order to analyze *TACSTD2*/Trop2 expression in healthy lungs, several databases were searched. First, the keyword “*TACSTD2*” was searched in every species included in the Expression atlas database^77^ (https://www.ebi.ac.uk/gxa/home, accessed on January 5, 2021). The results were filtered for baseline lung expression in each organism. From each study, information about expression level and the number of biological replicates was retrieved. The expression value was set to 0.5.

“*TACSTD2*” was also searched in The Human Protein Atlas^78^ (https://www.proteinatlas.org/, accessed on January 5, 2021) where the Tissue atlas was selected, and Lung was chosen to obtain RNA and protein expression data. For single cell analysis, dataset GSE130148 by Vieira Braga *et al.*^30^ containing single cell RNA sequencing analysis of fresh resected human lung tissue of uninvolved areas of tumor resection material from four patients was selected. Normalized transcript expression values for each cell clusters shown as nTPM (transcripts per million) were retrieved from the Human Protein Atlas (on April 26th, 2022). nTPM values were calculated as follows: the total read counts for all genes in each cluster was calculated by adding up the read counts of each gene in all cells belonging to the corresponding cluster. Next, the read counts were normalized to transcripts per million protein coding genes (pTPM) for each of the single cell clusters. TPM values of all samples within each data source were normalized separately using Trimmed mean of M values (TMM) to allow for between-sample comparisons. The resulting normalized transcript expression values, denoted nTPM, were calculated for each gene in every sample. To generate expression values per cell type, clusters were aggregated per cell type by first calculating the mean nTPM in all cells with the same cluster annotation within a dataset. The values for the same cell types in different data sets were then mean averaged to a single aggregated value.

“*TACSTD2*” was also searched in GTEx Portal^79^ (https://www.gtexportal.org/home/, accessed on January 5, 2021, dbGaP accession number: phs000424.v8.p2). Even though, data we used from GTEx are also available in Expression Atlas and The Human Protein Atlas, the GTEx is the primary source and data in other databases are not always up to date. Therefore, when data were available from multiple sources, we used data from GTEx for analyses.

Furthermore, “*TACSTD2*” was searched in Bgee database^80^ (https://bgee.org/, accessed on January 5, 2021). Because Bgee database uses ArrayExpress, GEO, and GTEx–dbGAP as sources of raw data, and the same sources are also used by the Expression Atlas, *TACSTD2* expression data from Bgee were used only for animals which *TACSTD2* expression profile was not listed in Expression Atlas database, i.e., pig, chimpanzee, macaque, rabbit, and opossum (see Supplementary File 1).

### Differential expression data analysis

To test the hypothesis that the overexpression of *TACSTD2* in the lungs is a physiological reaction to infection or injury, we first analyzed differential *TACSTD2* expression data for every species included in the Expression atlas database^77^ (https://www.ebi.ac.uk/gxa/home, accessed on January 5, 2021). Results were filtered by choosing Infect or Injury in Experimental variables. Subsequently, only studies that met the following criteria were included:

1. The dataset must contain data from experiments with whole organisms, not cell lines.
2. The dataset provides information about lung transcriptome.
3. The dataset allows identification of differentially expressed genes following infection or injury.

Additionally, the E-GEOD-33266 dataset, which was not found by the above-described approach as it did not contain the keyword “Infect” in its annotation, was identified by searching Expression Atlas COVID-19 Data Portal (www.covid19dataportal.org).

In each particular study, Log_2_-fold change value was set to 0.0 so we could see even the small change of *TACSTD2* expression. Microarray data were analysed as follows. Raw single-channel microarray intensities were normalized using RMA (robust multiarray avarage) via the oligo package from Bioconductor (Affymetrix data) or using quantile normalization via the limma package (Agilent data). Two-channel Agilent data were normalized using LOESS (locally estimated scatterplot smoothing) via the limma package. Pairwise comparisons are performed using *t*-test for each gene using limma. RNA-seq data was analysed using the iRAP pipeline. Quality-filtered reads were aligned to the latest version of the reference genome from Ensembl using TopHat2. Raw counts (number of mapped reads summarized and aggregated over each gene) were generated using htseq-count. Then, FPKM (fragments per kilobase of exon model per million mapped reads) and TPM were calculated. Pairwise comparisons were performed using a conditioned test based on the negative binomial distribution, using DESeq. p-values were adjusted for multiple testing using the Benjamini and Hochberg false discovery rate (FDR) correction. The adjusted p-value was set to 1, so we could see non-significant results as well^81^.

In order to pinpoint cell population(s) responsible for the increase of *TACSTD2* after infection in the lungs, we searched for datasets in Gene Expression Omnibus (GEO—www.ncbi.nlm.nih.gov/geo/)^82^ containing sorted cell populations from infected lungs or infected cell cultures *in vitro*. Datasets within GEO database contain data that are processed and normalized using a wide variety of methods, thereby the expression value for each dataset was specified in the Figure legends and corresponds to ‘Data processing’ field or VALUE description in the original sample records provided by a submitter. Expression values for *TACSTD2* from indicated datasets were retrieved, plotted and analyzed using GraphPad Prism v6.07 and shown in heat map using FGCZ Heatmap tool (http://fgcz-shiny.uzh.ch).

### Immunohistochemistry

The sample of human lungs was obtained from therapeutical surgery based on the written informed consent by the patients. The research was conducted in accordance with the Declaration of Helsinki and was approved by Ethics Committee of the University Hospital Brno (28-170621/EK). Mouse and pig lungs were collected in accordance with ARRIVE guidelines and the EU Directive 2010/63/EU. Animal experiments were were performed in accordance with relevant guidelines and regulations and approved by Masaryk University Institutional Animal Care and Use Committee. The specimens of pig, mouse and human lungs were fixed with formalin, washed with PBS, autotechnicon processed and embedded in paraffin blocks. Sections (4 μm thick) were dewaxed in xylene, hydrated through a graded series of alcohols (96%, 80%, and 70%), and rinsed in deionized water. After antigen retrieval in citrate buffer (pH 9.0) at 98 °C for 30 min., the slides were rinsed in tap and deionized water and washed with 3% H_2_O_2_ in PBS at room temperature (RT) for 10 min. To block endogenous peroxidase activity, the sections were treated with 10% fetal bovine serum for 30 min. The sections were incubated with the primary antibody against Trop2 (ab227689, 1:100, Abcam, Cambridge, United Kingdom) for 1 h at RT. The slides were then washed three times in PBS and subsequently incubated with the secondary antibody (En Vision FLEX/HRP, Dako, Agilent, Santa Clara, CA) for 20 min. After the last washing step, the slides were incubated in substrate solution (DAB), counterstained in hematoxylin, dehydrated with alcohols and xylene, and mounted.

### Generation of *TACSTD2* knock-out Calu-3 cells

Calu-3 cells were cultured in a humidified incubator (37 °C, 5% CO_2_) in Minimum Essential Medium (MEM) (Gibco, ThermoFisher Scientific, Waltham, MA) with 10% fetal bovine serum (FBS) (Invitrogen, ThermoFisher Scientific, Waltham, MA), 2 mM l-glutamine, 100 U/ml penicillin and 100 μg/ml streptomycin (Lonza, Basel, Switzerland). To generate *TACSTD2* KO cells, guide RNA (gRNA) sequences for CRISPR/Cas9 were designed by the CRISPOR online tool^83^. The 25-bp forward and reverse oligonucleotides (5ʹ-CACCGCGCCAGTGCAACCAGACGT-3ʹ, 5ʹ-AAACACGTCTGGTTGCACTGGCGC-3ʹ) comprising 20 bp *TACSTD2*-target sequence and *Bsm*BI sticky ends were annealed and inserted into the pSpCas9(BB)-2A-GFP plasmid^84^. Cells were transfected using Lipofectamine^TM^ 3000 (Invitrogen). Next day, GFP-positive cells were sorted at a dilution of 1 cell/well into a 96‐well plate on a FACSAria II Sorp 4L system (BD Biosciences, Franklin Lake, NJ). Single-cell colonies were expanded and the absence of Trop2 was verified by flow-cytometry as described previously^2^. Genomic DNA from these *TACSTD2* KO cells was isolated and short Ins/Del mutations within *TACSTD2* target sequence were confirmed by Sanger sequencing.

### Cell proliferation

1 × 10^5^ of control and *TACSTD2* KO Calu-3 cells were cultured in 6-well plates for 6 days and cells were counted every other day using the CASY cell counter (Roche, Basel, Switzerland). Medium was refreshed third day of cultivation.

### ALI culture

Calu-3 control and *TACSTD2* KO cells were seeded on 24-well Transwell PET membrane inserts with a 0.4 µm pore diameter (Corning Inc., Corning, NY) at a density of 1 × 10^5^ cells/insert in 200 µl of cell suspension in the apical compartment and 500 µl of medium in the basolateral compartment. After 3 days when cells reached confluence, the medium was removed from the apical compartment to create air–liquid interface and cells were cultured for another 11 days. The culture medium was changed every second day.

### FITC-dextran permeability assay

Medium in basolateral and apical compartment was replaced for phenol-red free growth medium and the medium in the apical part was enriched with 1 mg/ml FITC-dextran (4 kDa, Sigma, Merck, Darmstadt, Germany). Inserts were incubated for 3 h at 37 °C. Afterwards, samples of basolateral medium were taken for analysis of fluorescence in Fluostar Galaxy reader (BMG Labtech GmbH, Ortenberg, Germany) using 485/520 nm excitation/emission wavelengths in triplicate. The data were corrected for the fluorescence values of the pure medium. The translation of fluorescence data to FITC-dextran concentrations was based on a calibration curve.

## Supplementary Information

Supplementary Information.

## Data Availability

The raw data obtained and analyzed in this study are available from the corresponding author upon reasonable request.
