# Supplementary material for: TACSTD2 upregulation is an early reaction to lung infection
Source: Sci Rep. 2022 Jun 10;12:9583. doi: 10.1038/s41598-022-13637-9 (PMC9185727; doi:10.1038/s41598-022-13637-9)
Supplement: Supplementary file 1 — Supplementary Information. [file 41598_2022_13637_MOESM1_ESM.pdf]

Supplementary file 1: *TACSTD2* expression in lungs

<u>Human (*Homo sapiens*)</u>

Expression Atlas

https://www.ebi.ac.uk/gxa/about.html

Transcriptomics			
**ArrayExpress Accession number**	**Developmental stage**	**Expression level (median TPM)**	**Number of subjects**
E-MTAB-3358	fetal	98	1
	adult	138	1
E-MTAB-2836	-	228	5
E-MTAB 1733	-	222	5
E-MTAB-513	-	200	1
E-MTAB-4344	-	187	1

Proteomics		
**PRIDE or ArrayExpress Accession number**	**Expression level**	**Number of subjects**
PXD010154	46946	1
E-PROT-3	below cutoff	2
E-PROT-1	0.00005	1

GTEx Portal

https://www.gtexportal.org/home/gene/TACSTD2

**Data source**	**Expression level (median TPM)**	**Number of subjects**
GTEx Analysis Release V8 (dbGaP Accession phs000424.v8.p2)	118.7	578

The Human Protein Atlas

https://www.proteinatlas.org/ENSG00000184292-TACSTD2/tissue/lung

	**Expression level (median TPM)**	**Number of subjects**
HPA RNA Seq	99.9	9

<u>Mouse (*Mus musculus*)</u>

Expression Atlas

https://www.ebi.ac.uk/gxa/about.html

Transcriptomics			
**ArrayExpress Accession number**	**Developmental stage or strain (where different)**	**Expression level (median TPM)**	**Number of subjects**
E-MTAB-3579	embryonic day 12	below cutoff	1
	embryonic day 14	2	1
	embryonic day 15	2	1
	embryonic day 16	3	1
	embryonic day 17	8	1
	embryonic day 18	9	1
	neonate	11	5
	juvenile	19	2
	adult	20	1
E-MTAB-2801	CD1	68	1
	DBA/2J	86	1
	C57BL/6	93	1
E-GEOD-74747	-	111	1
E-MTAB-599	-	115	6
E-MTAB-8573	-	112	3

Proteomics		
**DOI**	**Expression level**	**Number of subjects**
10.1074/mcp.m112.024919	below cutoff	1

<u>Other organisms</u>

Expression Atlas - transcriptomics

https://www.ebi.ac.uk/gxa/about.html

Cattle (*Bos Taurus*)

ArrayExpress Accession number	Expression level (median TPM)	Number of subjects
E-MTAB-2798	119	3
E-MTAB-2596	84	1

Chicken (*Gallus gallus*)

ArrayExpress Accession number	Expression level (median TPM)	Number of subjects
E-MTAB-2797	2	3

Sheep (*Ovis aries*)

ArrayExpress Accession number	Sex and developmental stage	Expression level (median TPM)	Number of subjects
E-MTAB-3838	male, adult	17	1
	female, adult	9	1
	female, juvenile	18	1
E-GEOD-56643	-	66	1

Olive baboon (*Papio anubis*)

ArrayExpress Accession number	Expression level (median TPM)	Number of subjects
E-MTAB-2848	38	1

Rat (*Rattus norvegicus*)

ArrayExpress Accession number	Sex and developmental stage or strain (where different)	Expression level (median TPM)	Number of subjects
E-GEOD-53960	male, juvenile	31	4
	male, adolescent	42	4
	male, adult	49	4
	male, elderly	55	4
	female, juvenile	37	4
	female, adolescent	36	4
	female, adult	55	4
	female, elderly	57	4
E-MTAB-2800	F344/Cr1	121	1
	BN/SsNHsd	51	1
	Sprague-Dawley	97	1

Pig (*Sus scrofa*)

ArrayExpress Accession number	Sex	Expression level (median TPM)	Number of subjects
E-MTAB-5895	male	below cutoff	1
	female	below cutoff	1

Bgee database

https://bgee.org/

Organism	Expression score (median)	Number of subjects
Human (*Homo sapies*)	97.83	11
Mouse (*Mus musculus*)	72.83	26
Chimpanzee (*Pan troglodytes*)	68.31	1
Macaque (*Macaca mulata*)	92.61	1
Rat (*Rattus norvegicus*)	89.28	1
Cattle (*Bos taurus*)	78.34	1
Pig (*Sus scrofa*)	64.33	2
Rabbit (*Oryctolagus cuniculus*)	96.55	1
Opossum (*Monodelphis domestica*)	90.48	1
Chicken (*Gallus gallus*)	27.63	1

Supplementary file 2.

Immunohistochemical detection of Trop2 in paraffin sections of human, mouse, and pig lung tissue. Human lungs – positive staining in epithelium of airway and alveoli. Mouse/Pig lungs – positive staining only in basolateral parts of airway epithelium.

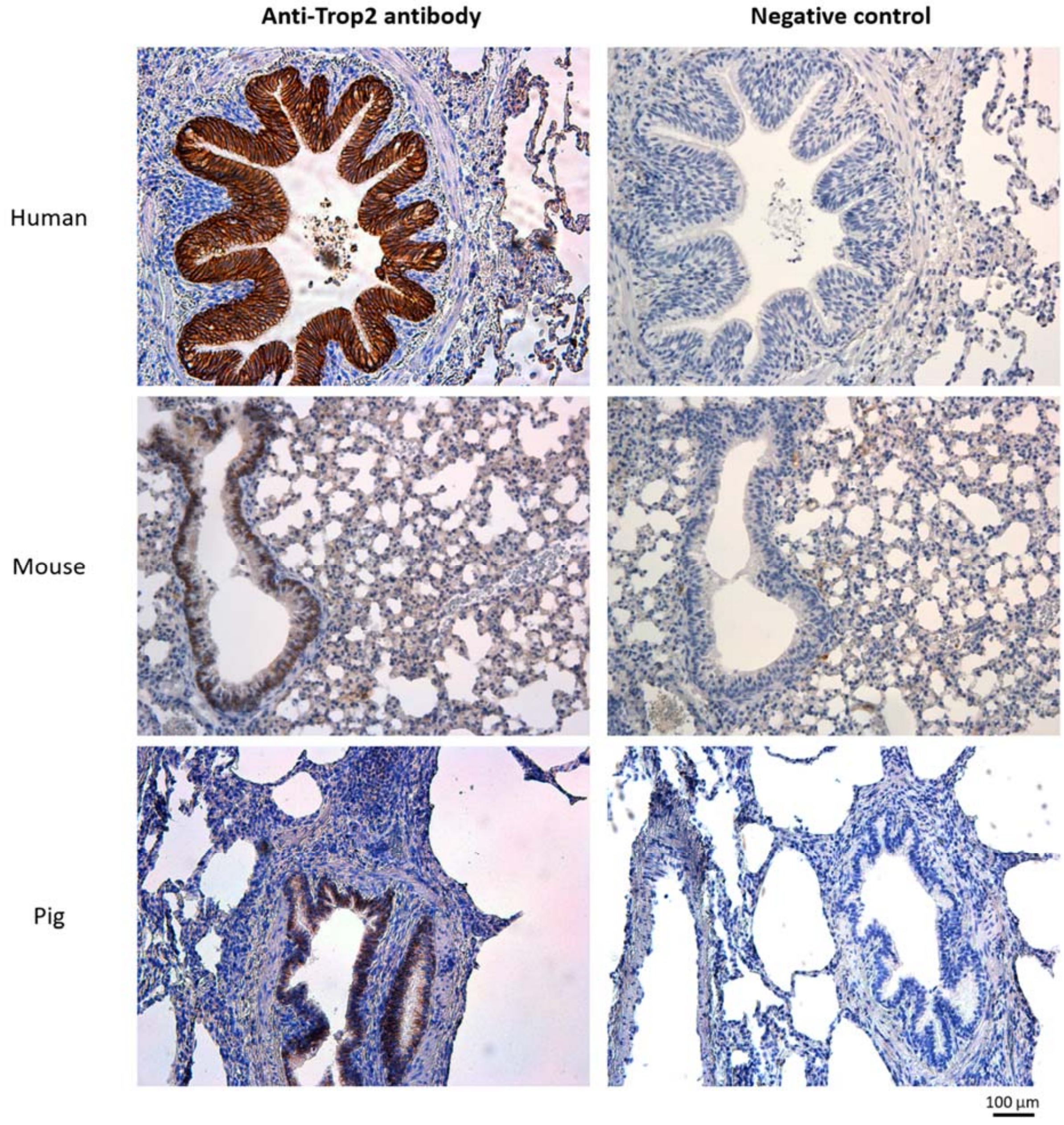

.

Supplementary file 3.

Immunohistochemical detection of Trop2 in paraffin sections of human lung tissues from 4 individuals.

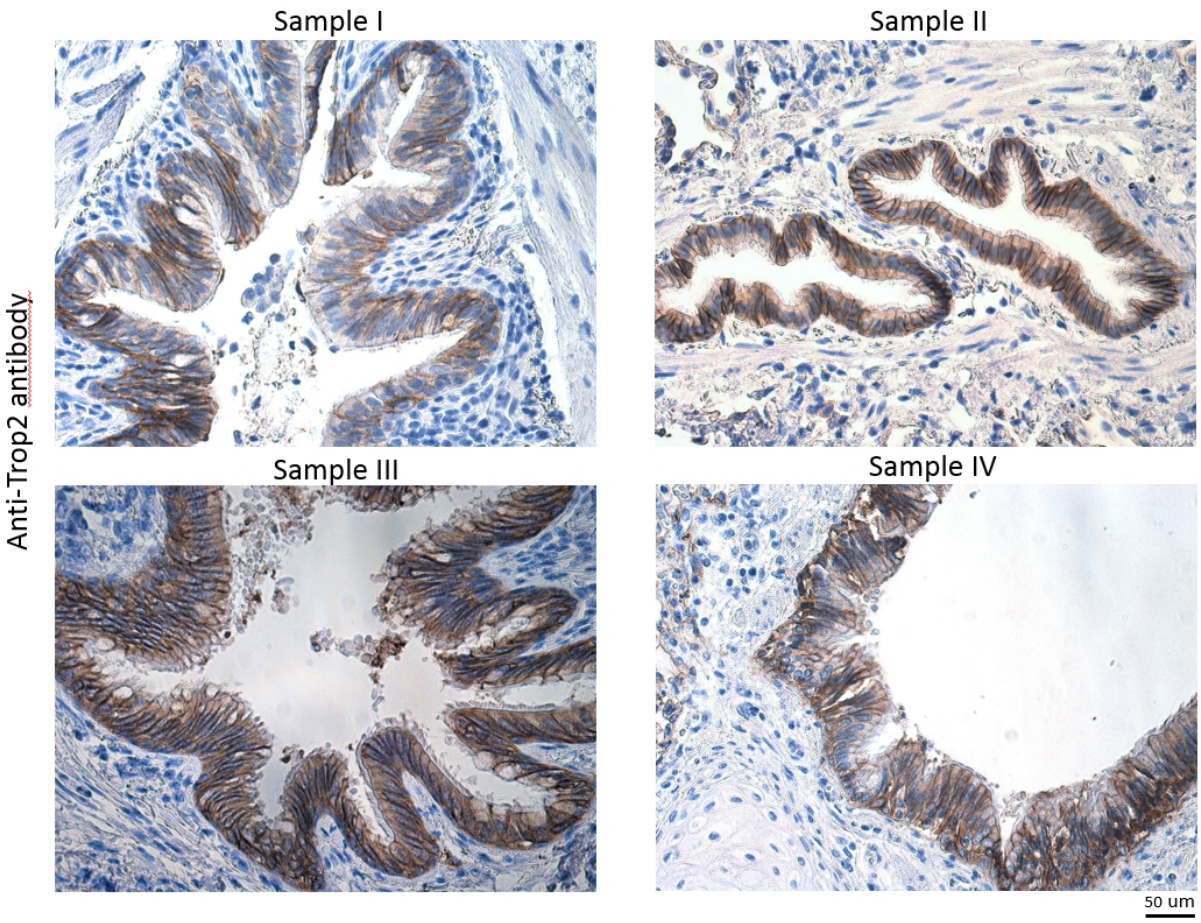

Supplementary file 4

Differential *TACSTD2* expression of bronchoalveolar lavage cells in patients with transplanted lungs colonized by *Aspergillus fumigatus* (E-MTAB-6040)

Infect	Log_2-fold change	Adjusted p-value	Number of subjects
Aspergillus fumigatus vs none	1.8	**0.028**	6 vs 5

Supplementary file 5

Differential *TACSTD2* expression of blood samples from pediatric and adults patients with burn injury (E-GEOD-19743). Samples were collected in early stage (<11 days) and middle stage (11-49 days) after injury.

	**Log_2-fold change**	**Adjusted p-value**	**Number of subjects**
Adult, early stage vs adult, control	1	**0.035**	29 vs 28
Adult, middle stage vs adult, control	3	**< 0.001**	28 vs 28
Child, early stage vs child, control	0.7	**0.038**	25 vs 35
Child, middle stage vs child, control	2.6	**< 0.001**	24 vs 35

Supplementary file 6

Growth and cell morphology in Calu-3 control and *TACSTD2* KO cells cultutred in ALI conditions. Cells were seeded on 24-well Transwell PET membrane inserts. After 3 days when cells reached confluence (day 0), the medium was removed from the apical compartment and cells were cultured for another 10 days. Cell morphology was observed by phase-contrast microscopy using the same magnification (100×).

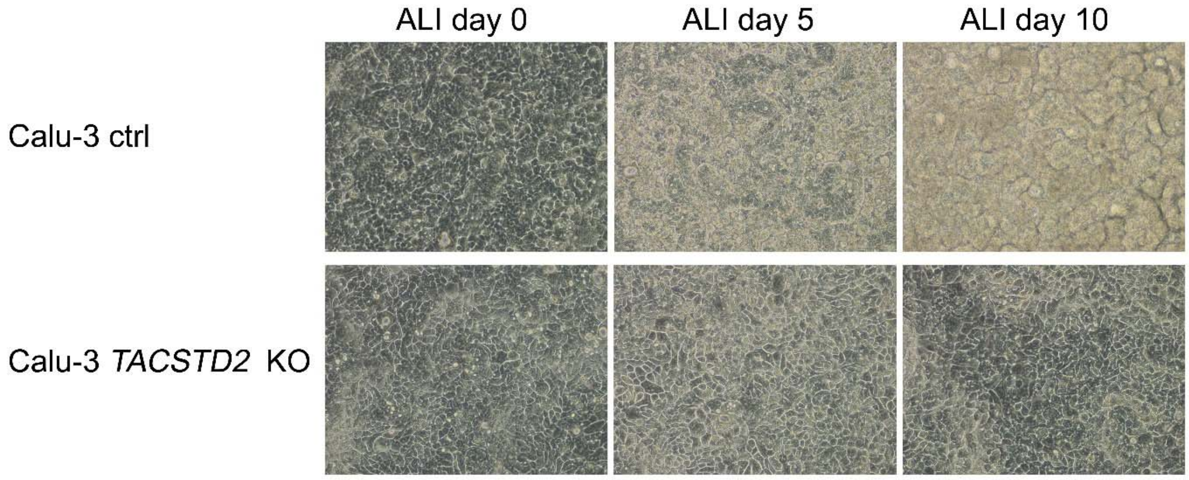

Supplementary file 7. Differential *Epcam* expression in mice after infection with various pathogens. Where not otherwise specified, viral infection dose was 10^5 plaque forming units (PFU). Significant results (adjusted p-value < 0.05) are labeled in bold. * means that this entry was missing from Expression Atlas and $\log_2$-fold change was calculated from GEO database data using GEO2R. N/A means that p-value could not be calculated due to small number of subjects or that change in *Epcam* expression was not analyzed in given dataset.

ArrayExpress accession number	Infect	Time (days)	Log_2-fold change	Adjusted p-value	Number of subjects	Strain	Age	Sex
E-GEOD-49262	SARS coronavirus MA15 dORF6 vs none	1	-0.3	**0.034**	3 vs 3	C57BL/6J	20 weeks	mixed
		2	0.2	0.145	3 vs 3			
		4	-0.1	0.783	3 vs 3			
		7*	-0.6	N/A	3 vs 2			
	SARS coronavirus MA15 vs none	1	-0.3	0.118	3 vs 3	C57BL/6J	20 weeks	mixed
		2	-	-	3 vs 3			
		4	0.3	0.352	3 vs 3			
		7*	-0.5	N/A	3 vs 2			
E-GEOD-49263	SARS coronavirus MA15 nsp16-/- vs none	1*	-0.4	N/A	3 vs 2	C57BL/6J	10 weeks	mixed
		2	0.4	**0.005**	4 vs 3			
		4	-0.3	0.132	3 vs 3			
		7	-0.6	**0.007**	4 vs 3			
	SARS coronavirus MA15 vs none	1*	-0.4	N/A	4 vs 2	C57BL/6J	10 weeks	mixed
		2	0.2	0.131	4 vs 3			
		4	-0.1	0.674	4 vs 3			
		7	-0.8	**< 0.001**	3 vs 3			
E-GEOD-50878	SARS coronavirus MA15 vs none	2	0.3	0.056	3 vs 9	C57BL/6J	10 weeks	not available
		4*	-0.3	N/A	2 vs 9			
		7	-0.4	**0.007**	3 vs 9			
	SARS coronavirus MA15 vs none	2	0.2	**0.016**	3 vs 7	C57BL/6J CXCR3 knockout	10 weeks	not available
		4*	0.4	N/A	2 vs 7			
		7	-0.7	**< 0.001**	4 vs 7			
E-GEOD-52405	SARS coronavirus MA15 vs mock	2	0.4	0.097	3 vs 4	129S1/SvImJ	8 to 16 weeks	female
		4	-	-	3 vs 4			

		2	0.3	0.094	3 vs 4	C57BL/6J	8 to 16 weeks	female
		4	0.3	0.067	3 vs 4			
		2	0.4	**0.039**	3 vs 4	CAST/EiJ	8 to 16 weeks	female
		4	0.5	**0.043**	3 vs 4			
		2	0.2	0.574	3 vs 4	NOD/ShiLtJ	8 to 16 weeks	female
		4	0.1	0.798	3 vs 4			
		2	0.5	**0.011**	3 vs 4	PWK/PhJ	8 to 16 weeks	female
		4	0.6	**< 0.001**	3 vs 4			
		2	0.3	0.297	3 vs 4	WSB/EiJ	8 to 16 weeks	female
		4	0.5	**0.013**	3 vs 4			
		4	0.3	0.191	3 vs 4	A/J	8 to 16 weeks	female
	influenza A virus (A/Puerto Rico/8/1934(H1N1)) (10^2 PFU) vs mock	2	0.4	0.085	3 vs 4	129S1/SvImJ	8 to 16 weeks	female
		4	0.3	0.149	3 vs 4			
		2	0.2	0.474	3 vs 4	A/J	8 to 16 weeks	female
		4	0.2	0.802	3 vs 4			
		2	0.9	**< 0.001**	3 vs 4	NOD/ShiLtJ	8 to 16 weeks	female
		4	0.3	0.068	3 vs 4			
		2	-0.1	0.93	3 vs 4	C57BL/6J	8 to 16 weeks	female
		4	0.5	**0.025**	3 vs 4			
		2	0.2	0.626	3 vs 4	NZO/HlLtJ	8 to 16 weeks	female
		4	0.5	0.24	3 vs 4			
		4	-	-	3 vs 4	PWK/PhJ	8 to 16 weeks	female
		2	0.3	0.232	3 vs 4	CAST/EiJ	8 to 16 weeks	female
		2	-	-	3 vs 4	WSB/EiJ	8 to 16 weeks	female
		4	0.7	**< 0.001**	3 vs 4			
E-GEOD-68820	SARS coronavirus MA15 vs mock	2	0.3	**0.006**	5 vs 4	C57BL/6NJ TLR3 knockout	10 weeks	female
		4	0.2	**0.022**	5 vs 4			
		7*	-0.02	N/A	5 vs 2			
		2	0.2	**0.034**	5 vs 5	C57BL/6NJ	10 weeks	female
		4	0.2	**0.015**	4 vs 5			
		7	-0.2	**0.014**	4 vs 4			

E-GEOD-59185	SARS coronavirus MA15 vs mock	2	N/A	N/A	3 vs 3	BALB/c	16 weeks	female
	SARS coronavirus MA15 E protein mutant Δ3 vs mock	2	N/A	N/A	3 vs 3	BALB/c	16 weeks	female
	SARS coronavirus MA15 E protein mutant Δ5 vs mock	2	N/A	N/A	3 vs 3	BALB/c	16 weeks	female
	SARS coronavirus MA15 lacking full-length E protein vs mock	2	N/A	N/A	3 vs 3	BALB/c	16 weeks	female
E-MTAB-5218	Mycobacterium tuberculosis H37Rv (1000 ± 300 CFU) vs none	28	-	-	4 vs 3	C57BL/6 TNF-α knock-out	8 to 12 weeks	female
		28	-0.2	**< 0.001**	10 vs 9	C57BL/6	8 to 12 weeks	female
E-GEOD-51386	SARS coronavirus MA15 (10^4 PFU) vs mock	4	0.1	0.347	4 vs 4	C57BL/6	20 weeks	not available
		7	-0.2	**0.006**	3 vs 4			
		4	0.2	0.185	4 vs 4	C57BL/6 PAI1 knockout	20 weeks	not available
		7	-0.3	**0.026**	3 vs 4			
		4	-0.1	0.405	4 vs 4	C57BL/6 TIMP1 knockout	20 weeks	not available
		7	-0.5	**< 0.001**	4 vs 4			
E-MTAB-6044	influenza A virus (500 PFU) vs mock (treatment with IgG1 isotype control)	7	N/A	N/A	4 vs 3	C57BL/6	8 to 10 weeks	male
	influenza A virus (500 PFU) vs mock (treatment with interleukin-22)	7	N/A	N/A	4 vs 4	C57BL/6	8 to 10 weeks	male
E-GEOD-51387	SARS coronavirus MA15 vs mock	4	0.2	**0.033**	3 vs 4	C57BL/6	20 weeks	not available
		7*	-0.4	N/A	2 vs 4			
		4*	-	-	2 vs 4	C57BL/6 PLAT knockout	20 weeks	not available
		7	-0.3	**0.006**	3 vs 4			
E-GEOD-10964	active Sendai virus vs UV-inactivated Sendai virus (Affymetrix MOE430A Array)	21	0.7	**0.005**	3 vs 3	C57BL/6J	3 to 5 weeks	male

	active Sendai virus vs UV-inactivated Sendai virus (Affymetrix Mouse430_2 Array)	49	0.7	**0.010**	3 vs 3	C57BL/6J	3 to 5 weeks	male
E-GEOD-40824	SARS coronavirus MA15 vs none	4	-0.1	0.675	3 vs 3	C57BL/6J	10 weeks	female
		7	-0.2	0.124	3 vs 3			
		4	-	-	3 vs 3	C57BL/6J Tnfrsf1a/1b knockout	10 weeks	female
		7*	-0.5	N/A	2 vs 2			
E-GEOD-33266	SARS coronavirus MA15 (10^2 PFU) vs none	1	0.3	**0.040**	5 vs 3	C57BL/6	20 weeks	female
		2	0.5	0.237	5 vs 3			
		4	0.1	0.382	5 vs 3			
		7	-	-	5 vs 3			
	SARS coronavirus MA15 (10^3 PFU) vs none	1	0.4	**0.009**	5 vs 3	C57BL/6	20 weeks	female
		2	0.2	0.444	5 vs 3			
		4	0.1	0.506	5 vs 3			
		7	-0.3	**0.020**	5 vs 3			
	SARS coronavirus MA15 (10^4 PFU) vs none	1	0.6	**0.006**	5 vs 3	C57BL/6	20 weeks	female
		2	0.2	0.408	5 vs 3			
		4	0.3	0.080	5 vs 3			
		7	-0.3	**0.013**	5 vs 3			
	SARS coronavirus MA15 vs none	1	0.6	**0.020**	5 vs 3	C57BL/6	20 weeks	female
		2	0.3	0.060	5 vs 3			
		4	0.1	0.455	5 vs 3			
		7	-0.3	**0.017**	5 vs 3			
